# Rewiring of regenerated axons by combining treadmill training with semaphorin3A inhibition

**DOI:** 10.1186/1756-6606-7-14

**Published:** 2014-03-10

**Authors:** Liang Zhang, Shinjiro Kaneko, Kaoru Kikuchi, Akihiko Sano, Miho Maeda, Akiyoshi Kishino, Shinsuke Shibata, Masahiko Mukaino, Yoshiaki Toyama, Meigen Liu, Toru Kimura, Hideyuki Okano, Masaya Nakamura

**Affiliations:** 1 Department of Orthopedic Surgery, Keio University School of Medicine, 35 Shinanomachi, Shinjuku, Tokyo 160-8582, Japan; 2 Department of Physiology, Keio University School of Medicine, 35 Shinanomachi, Shinjuku, Tokyo 160-8582, Japan; 3 Department of Rehabilitation Medicine, Keio University School of Medicine, 35 Shinanomachi, Shinjuku, Tokyo 160-8582, Japan; 4 Department of Orthopedic Surgery, National Hospital Organization, Murayama Medical Center, 2-37-1 Gakuen, Musashimurayama, Tokyo 208-0011, Japan; 5 Dainippon Sumitomo Pharma Co. Ltd., 3-1-98 Kasugade-naka, Konohana-ku, Osaka 554-0022, Japan

**Keywords:** Axonal regeneration, Semaphorin3A, Inhibitor, Rehabilitation, Rewiring, Drug delivery system

## Abstract

**Background:**

Rats exhibit extremely limited motor function recovery after total transection of the spinal cord (SCT). We previously reported that SM-216289, a semaphorin3A inhibitor, enhanced axon regeneration and motor function recovery in SCT adult rats. However, these effects were limited because most regenerated axons likely do not connect to the right targets. Thus, rebuilding the appropriate connections for regenerated axons may enhance recovery. In this study, we combined semaphorin3A inhibitor treatment with extensive treadmill training to determine whether combined treatment would further enhance the “rewiring” of regenerated axons. In this study, which aimed for clinical applicability, we administered a newly developed, potent semaphorin3A inhibitor, SM-345431 (Vinaxanthone), using a novel drug delivery system that enables continuous drug delivery over the period of the experiment.

**Results:**

Treatment with SM-345431 using this delivery system enhanced axon regeneration and produced significant, but limited, hindlimb motor function recovery. Although extensive treadmill training combined with SM-345431 administration did not further improve axon regeneration, hindlimb motor performance was restored, as evidenced by the significant improvement in the execution of plantar steps on a treadmill. In contrast, control SCT rats could not execute plantar steps at any point during the experimental period. Further analyses suggested that this strategy reinforced the wiring of central pattern generators in lumbar spinal circuits, which, in turn, led to enhanced motor function recovery (especially in extensor muscles).

**Conclusions:**

This study highlights the importance of combining treatments that promote axon regeneration with specific and appropriate rehabilitations that promote rewiring for the treatment of spinal cord injury.

## Background

Severe spinal cord injuries (SCI) in adult mammals result in various deficits throughout life. The limited capability of axons to regenerate in the central nervous system (CNS) is thought to be the main reason for these lasting deficits. Previous studies have suggested that both extrinsic and intrinsic factors in the CNS contribute to this incapacity for axonal regeneration [1-4]. Several distinct extrinsic molecules have been proposed to hinder axonal regeneration, including CNS myelin-associated proteins (MAG, Nogo, OMgp) [5-9], chondroitin sulphate proteoglycans [10,11], semaphorin3A [12,13] and RGM (repulsive guidance molecule) [14,15]. Neutralizing one (or several) of these molecules enhances axonal regeneration and results in some degree of functional recovery [10,16,17]. Until recently, it remained unknown whether neutralizing semaphorin3A would also lead to axonal regeneration and motor function recovery, in part because semaphorin3A deficiency is lethal [18]. Thus, we previously developed a selective and potent semaphorin3A inhibitor called SM-216289 [19] that selectively inhibits semaphorin3A signaling both *in vitro* and *in vivo*[20]. Administration of SM-216289 to adult rats after total spinal cord transection (SCT) led to axonal regeneration and motor function recovery [20]. In addition, axonal regeneration and functional recovery have now been observed after several treatments that block 1 or more axonal growth inhibitors (including SM-216289). However, these effects are moderate at best, presumably because most of the regenerated axons do not connect with the correct targets [21]. Thus, rebuilding the appropriate connections of regenerated axons in lesioned spinal cords remains an important unresolved issue.

Body weight-supported treadmill training induces plastic changes in lesioned spinal cords and is useful for maximizing residual locomotor function after moderate SCI [22,23]. Furthermore, even after severe SCI, treadmill training partially improves hindlimb coordination [24] by inducing plasticity in specific spinal locomotor circuits called “central pattern generators” (CPGs). More specifically, these plastic changes have been shown to result in the recovery of plantar step walking in cats [25] and neonatal rats [26]. Furthermore, SCT adult rats partially recover plantar step walking when treadmill training is combined with other appropriate treatments, such as epidural electrical stimulation [27], pharmacological treatments [24] or cell transplantation [28]. Thus, with specific and appropriate rehabilitation, spinal cord CPGs can be reorganized, and functionally appropriate connections between CPGs and regenerated (or residual) axons can be rebuilt. Therefore, we hypothesized that extensive treadmill training would assist in the correct wiring of axons regenerated by semaphorin3A inhibitor treatment and that this rewiring may contribute to further motor functional recovery after SCT.

However, several issues, including drug delivery, remain to be resolved before semaphorin3A inhibitors can be used in the clinic. In an attempt to resolve these issues, we developed a novel selective semaphorin3A inhibitor, SM-345431 (Vinaxanthone), which demonstrates physicochemical properties equivalent to those of SM-216289 but also improvements that should allow for the development of a higher quality pharmaceutical product. Additionally, we developed a novel drug delivery system (DDS) utilizing a silicone sheet. With future clinical applications in mind, we chose to evaluate SM-345431 with this novel DDS. We observed that, consistent with our previous study [20], SM-345431 treatment enhanced axon regeneration and resulted in significant, but limited, hindlimb motor function recovery. Although extensive treadmill training with SM-345431 administration did not further improve axon regeneration, hindlimb motor performance was restored, as evidenced by the execution of plantar steps on a treadmill using a body support system (BSS). Moreover, immunohistological analysis suggested that SM-345431 administration with treadmill training reinforced the wiring of CPGs in lumbar spinal circuits and led to enhanced motor function recovery, especially in extensor muscles.

## Results

### Evaluation of a novel DDS and the activity of SM-345431 *in vitro*

**Figure 1:**
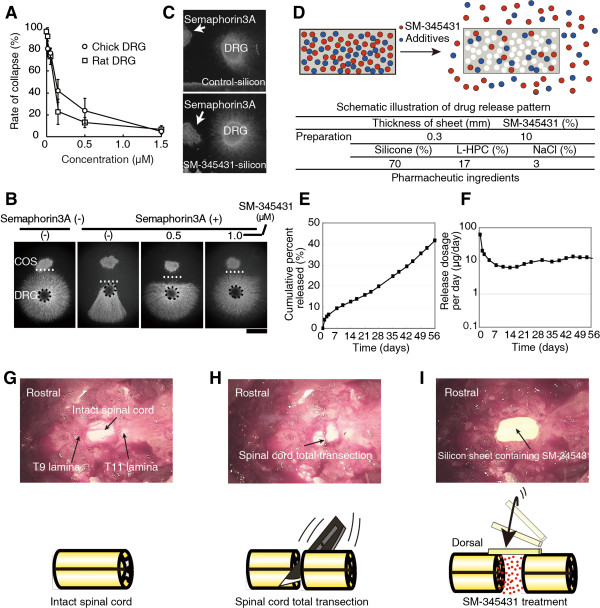
**Preparation of the SM-345431 silicone DDS and *in vitro *release study. (A)** Analysis of the inhibitory activity of SM-345431 in the growth cone collapse assay using E8 chick and E14 rat DRGs. **(B)** Collagen co-culture assay using E8 chick DRGs and semaphorin3A-expressing COS7 cell aggregates. **(C)** Pictures showing the effects of SM-345431-silicone or control-silicone in the collagen gel. **(D)** Schematic illustration of drug release from the matrix silicone preparation; the drug is dissolved in water, permeates into the preparation and is then released. The rate of water permeation into the silicone matrix is controlled by various additives, and the proportions of these additives included in the DDS control the variations in the drug release profile. The preparation used here contained the water-soluble additives listed in the table **(D)**. Stable and linear release of SM-345431 was observed for up to 2 months as shown in **(E)** and **(F)**. **(E**, **F)** SM-345431 release curve for the preparation. The linear cumulative percent release curve at 2 months **(E)** and the stable release dosage per day **(F)** are shown for the administration of SM-345431 using this novel DDS. **(G)** Exposed intact spinal cord. **(H)** Transected spinal cord. **(I)** The novel DDS we employed. A silicone sheet containing SM-345431 was placed onto the transected site of the spinal cord. The system allowed for stable and continuous release of the semaphorin3A inhibitor throughout the experimental period.

In our previous study, we used an osmotic mini-pump to deliver the semaphorin3A inhibitor SM-216289 [20]. However, in clinical practice, this type of invasive drug delivery method is not ideal. Therefore, we developed a novel DDS that utilizes a silicone matrix to continuously deliver SM-345431 (a newly developed semaphorin3A inhibitor) intrathecally. We evaluated the drug release profile of SM-345431 in this new silicone matrix preparation and the potency of SM-345431-mediated semaphorin3A inhibition in vitro (Figure 1). SM-345431 exhibited semaphorin3A inhibiting activity with an IC50 of 0.1-0.2 μM in growth cone collapse assays using E8 chick and E14 rat dorsal root ganglia (DRG) (Figure 1A). When chick embryonic DRG explants and semaphorin3A-expressing COS7 cell aggregates (semaphorin3A-COS) were co-cultured in a collagen gel, the neurites of the DRG explants grew away from the semaphorin3A-COS, as shown in Figure 1B. However, when DRG explants and semaphorin3A-COS were co-cultured in the presence of SM-345431, radial extensions of the neurites were observed, which suggests that the chemo-repulsive effects of semaphorin3A were blocked by SM-345431 in a dose-dependent manner (Figure 1B). We also evaluated the selectivity of SM-345431 for semaphorin3A inhibition by examining the pharmacological profile of SM-345431 (Tables 1 and 2). As shown in these tables, the IC50 value for semaphorin3A inhibition was substantially lower than the other IC50s, which suggested that SM-345431 is a highly selective semaphorin3A inhibitor. To examine the semaphorin3A inhibiting activity of SM-345431 while it was being released from the silicone matrix (SM-345431-silicone), 1 mg of a silicone sheet containing 100 μg SM-345431 was placed into collagen gel cultures containing DRG explants and semaphorin3A-COS (Figure 1C). Assuming that 5% of the SM-345431 was released and uniformly diffused throughout the culture during the 2 days of incubation, the final concentration of SM-345431 was approximately 5 μM, which is a large enough dose to inhibit semaphorin3A activity. Radial neurite extension was observed in cultures with SM-345431-silicone but not in those with control silicone, indicating that semaphorin3A activity had been inhibited by SM-345431. We also measured the cumulative percentage of released doses of SM-345431 using this DDS over 2 months *in vitro* (Figure 1E) and found that this DDS released a constant dose of SM-345431 and was stable *in vitro*. When 7 mm × 5 mm × 0.3 mm sheets were used, the amount of drug release stabilized at approximately 10 μg/day after an initial peak of drug release that occurred over the first 2 days (Figure 1F). For the *in vivo* study, we trimmed the silicone sheet into 3 mm × 3 mm × 0.3 mm pieces to fit the injury site following SCT (Figure 1G-I). The release of SM-345431 (0.1 mg/mg loading 10%) *in vivo* was calculated as 0.5-0.7 μg/day, and this dose was similar to the dose of the semaphorin3A inhibitor (SM-216289) [19] that we administered using osmotic mini pumps in our previous study [20]. Therefore, the newly developed DDS allowed stable and continuous release of the newly developed, potent semaphorin3A inhibitor SM-345431.

**Table 1:** Pharmacological profile of SM-345431 (part 1)

**Enzymes**	**IC50 (μm)**
Semaphorin	0.1-0.2
Matrix Metalloproteinase-1 (MMP-1)	>10
Matrix Metalloproteinase-7 (MMP-7)	10
Matrix Metalloproteinase-2 (MMP-2)	>10
Matrix Metalloproteinase-3 (MMP-3)	>10
Matrix Metalloproteinase-9 (MMP-9)	>10
Phospholipase PLA2-1	>10
Phospholipase PLC	>10
Caspase 1	>10
Caspase 3	>10
Caspase 6	>10
Caspase 7	>10
Caspase 8	>10
Protein Tyrosine Phosphatase, CD45	>10
Protein Tyrosine Phosphatase, PTP1B	>10
Protein Tyrosine Phosphatase, PTP1C	>10
Protein Tyrosine Phosphatase, T-Cell	>10
Sphingomyelinase, Neutral (N-SMase)	>10
Chemokine CCR1	>10
Chemokine CCR2B	>10
Chemokine CCR4	>10
Chemokine CCR5	>10
Chemokine CXCR2 (IL-8B)	>10
Glucocorticoid	>10
Interleukin IL-1	>10
Interleukin IL-2	>10
Interleukin IL-6	>10
Tumor Necrosis Factor (TNF), Non-selective	>10
Adhesion, fibronectin-mediated	>10
Adhesion, ICAM-1-Mediated	>10
Adhesion, VCAM-1-Mediated	>10
Cell proliferation, B-Cell+LPS	>10
Cell proliferation, T-Cell+Con A	>10
Mediator release, IL-1beta	>10
Mediator release, IFN-gamma	>10
Mediator release, IL-10	>10
Mediator release, IL-2	>10
Mediator release, IL-4	>10
Mediator release, IL-5	>10
Mediator release, IL-6	>10
Mediator release, TNF-alfa, PBML	>10
Transcription response, NF-AT	>10
Transcription response, NF-kB	>10

Summary of the IC50 values for binding assays of various receptors and ion channels, and IC50 values for the inhibition of various enzymes. The IC50 value for semaphorin3A inhibition was extremely low compared to that of the other factors.

**Table 2:** Pharmacological profile of SM-345431 (part 2)

**Kinases**	**IC50 (μm)**
CaMKII	>10
CDK5/p35	>10
cSRC	>10
EGFR	0.90
EphA2	>10
EphA4	0.80
EphB2	0.68
EphB4	>10
Fes	>10
FGFR1	>10
FGFR2	>10
FGFR3	0.77
FGFR4	0.64
Flt1	>10
Flt3	>10
Fyn	>10
GSK3α	2.46
GSK3β	>10
IGF-1R	>10
JAK3	>10
KDR	>10
MAPK2	>10
MEK1	>10
MEK4	>10
MKK6	>10
PAK2	>10
PAK4	>10
PKA	>10
PKBα	>10
PKBβ	>10
PKCγ	>10
ROCK-I	>10
ROCK-II	>10
ROCK-II	>10
SAPK2a	>10
TrkA	>10
TrkB	>10
PI 3-Kγ	>10

Summary of the IC50 values revealed by inhibition tests for various kinases. The data in Tables 1 and 2 suggest that SM-345431 was highly selective for semaphorin3A inhibition.

### SM-345431 delivery via the novel DDS enhanced axonal regeneration

**Figure 2:**
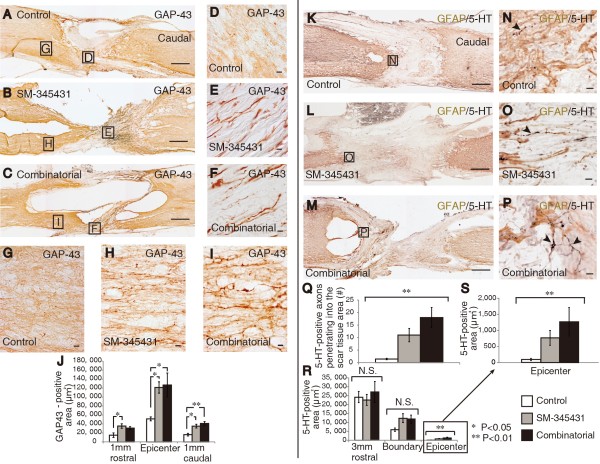
**SM-345431 enhanced axonal regeneration *in vivo*, but combined treatment had only a limited effect on further axon regeneration. (A-I)** Sagittal sections from SCT rats immunostained for GAP-43. Low-magnification images of the control **(A)**, SM-345431 **(B)** and combined treatment **(C)** groups. Scale bars = 500 μm. **(D-F)** Magnified images of the boxed areas shown in **A**-**C**. Scale bars = 10 μm. **(G-I)** Additional magnified images of the boxed areas shown in **A**-**C**. Scale bars = 10 μm. **(J)** Quantitative analysis of GAP-43-positive areas at 1 mm rostral to the lesion, 1 mm caudal to the lesion and at the epicenter of the lesion. Immunohistochemistry was performed using DAB with nickel enhancement. *P < 0.05, **P < 0.01. Statistical analyses were performed using one-way ANOVA and Bonferroni post hoc tests. Data are represented as the mean ± S.E.M. **(K-P)** Sagittal sections of SCT rats double-stained for 5-HT and GFAP. Scar tissue is outlined by GFAP staining, which also allowed for confirmation of total transection of the spinal cord in each animal. **(K-M)** Low-magnification images of the control **(K)**, SM-345431 **(L)** and combined treatment **(M)** groups. Scale bars = 500 μm. **(N-P)** Magnified images of the boxed areas shown in **K**-**M**. Scale bars = 10 μm. Arrowheads represent 5-HT-positive (serotonergic) axons. **(Q-S)** Quantitative analyses of 5-HT-positive axons that penetrated into the scar tissue. **(Q)** Quantitative analysis of the number of 5-HT-positive axons that penetrated into the lesion site. **(R,S)** Quantitative analysis of the 5-HT-positive area within the scar tissue area. Immunohistochemistry was performed by double-staining using DAB with or without nickel enhancement. The left side is rostral in all images. **P < 0.01. Statistical analyses were performed using a Kruskal-Wallis H test. Data represent the mean ± S.E.M.

To examine the regeneration of axons after SM-345431 treatment and SM-345431 treatment combined with extensive treadmill training, we evaluated axons in the injured spinal cord with immunostaining using antibodies against GAP43 and serotonin (5-HT) (Figure 2), GAP43 is widely used as a marker for regenerated axons. In both treatment groups, a marked increase in the number of GAP43-positive axons was observed at the epicenter of the injury (Figure 2D-F) and in the surrounding area (Figure 2G-I). Compared with the control group, the number of GAP43 axons was significantly increased in both the SM-345431 treatment group and the combined treatment group, especially at 1 mm caudal to the injury epicenter (Figure 2J). No significant difference was observed between the 2 treatment groups. Thus, administration of the semaphorin3A inhibitor SM-345431 using this DDS enhanced axonal regeneration. However, no additional axonal regeneration was observed when SM-345431 treatment was combined with treadmill training.

**Figure 3:**
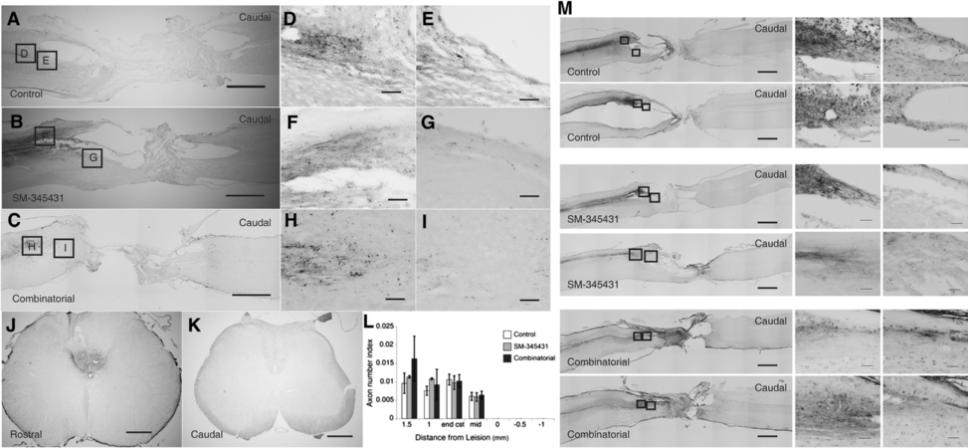
**Histological analyses of the treatment effects on CST regeneration in the injured spinal cord using BDA staining. J** shows a representative axial section of the spinal cord at the C4 level (rostral to the lesion site), and **K** shows a representative image of a section at the L4 level (caudal to the lesion site) (Scale bars = 500 μm). Based on these sections, we confirmed that the labeling efficiency of BDA tracing in each animal was sufficient. **A**, **B** and **C** show representative sagittal sections of the BDA staining in each group. **D**/**E** show magnified images of the boxed area in **A**. Similarly, **F**/**G** show magnifications of the areas in **B**, and the images in **H**/**I** are magnifications of the areas in **C**. Scale bars = 1,000 μm in **A**-**C** and 100 μm in **D**-**I**. No obvious CST regeneration beyond the lesion epicenter was observed in any of the groups examined **(L)**. Statistical analyses were performed using one-way ANOVA and Bonferroni *post hoc* analyses. Data are presented as the mean ± S.E.M. The images in **M** are representative of other sagittal sections from each group. Scale bars = 1,000 μm in the images on left side of **M** and 100 μm in the magnified images of **M**.

The raphespinal tract axons, which can be detected by immunohistochemistry against serotonin (5-HT), contribute to functional locomotor control, and regeneration of these axons leads to substantial enhancement of motor function recovery [28]. Therefore, we also evaluated the regeneration of raphespinal tract axons using a GFAP antibody to delineate scar tissue at the injury site and a 5-HT antibody to visualize raphespinal axons. In control animals, 5-HT-positive axons were restricted to the area rostral to the transected site, and few 5HT-positive axons entered the GFAP-negative scar tissue area (Figure 2K,N). Interestingly, significantly more 5-HT-positive axons penetrated the GFAP-negative scar tissue area after SM-345431 treatment and combined treatment as compared to the control conditions (Figure 2K-S). Because we used a total transection model in this study, the 5-HT-positive axons that penetrated the GFAP-negative scar tissue in the treatment groups were regarded as regenerated axons (Figure 2L-P). Cortico-spinal tract (CST) axons are known to be incapable of regeneration after transection, even following any of the previously reported treatments [7]. We next evaluated the regeneration of CST axons using the anterograde tracer biotinylated dextran amine (BDA). We first confirmed sufficient labeling efficiency of BDA tracing in each animal, and no obvious CST regeneration beyond the lesion epicenter was observed in either the control or the semaphorin3A inhibitor treatment group, which is consistent with our previous paper’s findings [20] (Figure 3). Moreover, no clear CST regeneration beyond the lesion epicenter was observed in the combined treatment group (Figure 3).

### SM-345431 enhanced angiogenesis and remyelination

**Figure 4:**
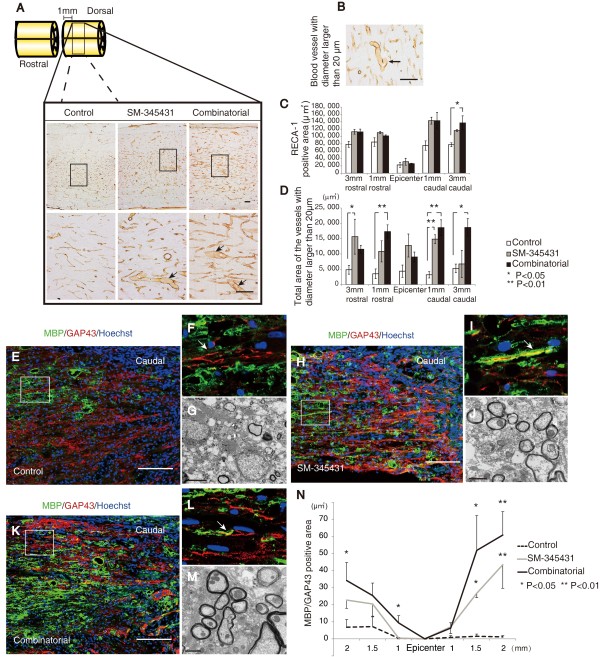
**Histological analyses of the treatment effects on microvasculature and remyelination in the spinal cord. (A)** Visualization of blood vessels using an anti-RECA-1 antibody. Images in the upper row are low-magnification views of the gray matter areas of sagittal sections immunostained for RECA-1 at 1 mm caudal to the transected site. Scale bars = 50 μm. Images in the lower row are high-magnification views that correspond to the boxed areas in the upper row images. Scale bars = 50 μm. **(B)** Representative image of a blood vessel with a lumen with a diameter larger than 20 μm (arrow), which indicated newly formed blood vessels following injury. Scale bars = 50 μm. Arrows in **(A)** also represent blood vessels with lumen diameters larger than 20 μm. The left side is rostral **(A,B)**. **(C)** Quantitative analysis of RECA-1-positive areas in each group. **(D)** Quantitative analysis of the total areas of RECA-1-positive blood vessels with lumen diameters larger than 20 μm. *P < 0.05, **P < 0.01. Statistical analyses were based on one-way ANOVA and Bonferroni post hoc analyses. **(E-M)** Analyses of remyelination performed using immunohistochemistry against MBP or electron microscopy 12 weeks post-injury. **(E,F,H,I,K,L)** Reconstructed confocal images showing double staining (sagittal sections) for MBP (green) and GAP43 (red) in the control group **(E**,**F)**, SM-345431 treatment group **(H**,**I)** and combined group **(K, L)**. **F**, **I** and **L** show magnified images of the boxed areas in **E**, **H** and **K**, respectively. Scale bars = 100 μm. The arrow in **F** shows a non-myelinated (MBP-negative) GAP-43-positive axon, and the arrows in **I** and **L** show myelinated (MBP-positive) GAP-43-positive axons. The left side is rostral. **(G,J)** Electron microscopic images of transverse sections from the control group **(G)** and SM-345431 treatment group **(J)** at the lesion site. Scale bars = 2 μm. **(N)** Statistical analysis of the number of myelinated (MBP-positive) GAP-43-positive axons in each group, which were analyzed by immunohistochemistry. *P < 0.05, **P < 0.01. Statistical analyses were performed using one-way ANOVA and Bonferroni *post hoc* analyses. All the data are represented as the mean ± S.E.M.

Semaphorin3A suppresses VEGF-induced angiogenesis, and inhibition of semaphorin3A leads to enhancement of angiogenesis [29]. This phenomenon occurs because semaphorin3A and VEGF share the same receptor, neuropilin1 [30]. In addition, blood vessels are believed to play important roles in tissue repair and axonal regeneration after SCI [31-33]. Therefore, we analyzed the effects of SM-345431 treatment (using our DDS) on angiogenesis. For immunohistochemistry, we used the anti-RECA-1 antibody, which is known to enable visualization of blood vessels and migrating endothelial cells in rats [32] (Figure 4A). RECA-1-positive areas 3 mm caudal to the lesion epicenter were significantly increased after combined treatment (Figure 4C, P < 0.05). Based on their morphology, thick-walled blood vessels with lumen diameters larger than 20 μm are thought to be newly formed blood vessels [34] (Figure 4A,B, arrows). Consistent with previous reports [34,35], these thick-walled blood vessels were rarely observed in the intact spinal cord. In comparison to the control group, the total immunostained areas of vessels with lumen diameters larger than 20 μm were significantly increased in both the SM-345431 and combined treatment groups 3 mm rostral/caudal and 1 mm rostral/caudal to the lesion epicenter (Figure 4D). Furthermore, the effects of angiogenesis tended to be enhanced in the combined treatment group compared to the SM-345431 treatment group, but this difference did not reach statistical significance (Figure 4C,D). Thus, SM-345431 treatment significantly increased the number of newly formed blood vessels.

Semaphorin3A also inhibits oligodendrocyte precursor cell recruitment and influences remyelination [36]. Using immunohistochemistry and electron microscopy, we next characterized the axons at the lesion sites after SM-345431 treatment in greater detail. In the SM-345431 treatment group, we observed substantial numbers of myelinated GAP43-positive axons at the lesion site (Figure 4H-J), whereas myelinated GAP43-positive axons were rarely observed at the lesion site in the control SCT group (Figure 4E-G). Based on their morphologies, the thin myelin sheathes observed in the SM-345431 group were likely the result of remyelination (Figure 4H-J). While SM-345431 treatment significantly enhanced the remyelination of axons (Figure 4N), additional remyelination was not observed in the SM-345431 plus treadmill training combined group (Figure 4K-N).

### Combining SM-345431 with treadmill training reinforced specific spinal locomotor circuitry and synaptic connectivity

**Figure 5:**
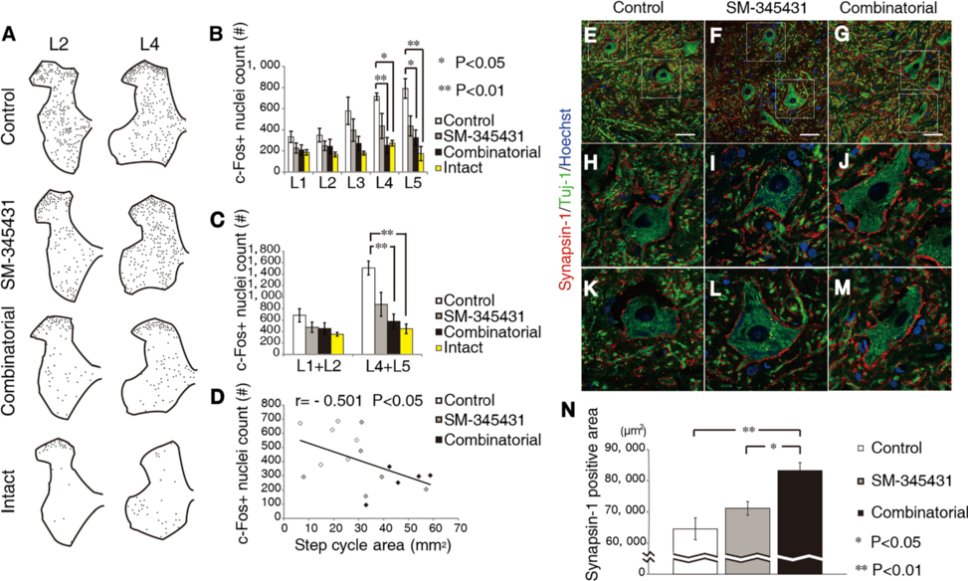
**Immunohistochemical analyses of the effects of treatment on functional remodeling of spinal circuits and synaptic connectivity at the lumbar enlargement. (A)** Representative Camera Lucida drawings of c-Fos + nuclei in transverse sections at the L2 and L4 levels from control rats, SM-345431 treatment rats, combined treatment rats and intact rats. **(B)** Average total numbers of c-Fos + nuclei at each level of the lumbar spinal segment. **(C)** Average total number of c-Fos + nuclei in rostral (L1 + L2) and caudal (L4 + L5) lumbar enlargement segments. **(D)** Correlation analysis between the total number of c-Fos + nuclei in all segments of the lumbar enlargement and step ability on the treadmill. SM-345431 treatment produced a trend toward decreases in the average number of c-Fos + nuclei, although this trend was not statistically significant compared to the control and SM-345431 treatment groups. However, the combined treatment produced a statistically significant decrease in the number of c-Fos + nuclei, suggesting that specific spinal locomotor circuitry was reinforced by combining SM-345431 treatment with extensive treadmill training. *P < 0.05, **P < 0.01. Statistical analyses were performed using one-way ANOVA and Bonferroni post hoc analyses. Data are presented as the mean ± S.E.M. **(E-M)** Representative reconstructed confocal images of double staining (transverse section) for synapsin-1 and Tuj-1 in the ventricolumnar area of the spinal cord (**E**-**G**: low-magnification images, **H**-**M**: high-magnification images). **H-J** and **K-M** show the upper and lower boxed areas in **E-G**, respectively. Scale bars = 50 μm. **(N)** Quantitative analysis of synapsin-1 expression in the ventricolumnar motor neuron area 3 months after SCT. *P < 0.05, **P < 0.01. Statistical analyses were performed using one-way ANOVA and Bonferroni *post hoc* analyses. Data are presented as the mean ± S.E.M.

Functional locomotor recovery in spinal cord models is critically dependent on supraspinal connections to CPGs [37]. To examine the combined effects of SM-345431 treatment and treadmill training on the reconstruction of spinal cord circuitry at the lumbar level, we performed immunostaining for c-Fos [38] and synapsin-1 [39,40]. c-Fos is widely used as a marker to measure the extent of the supraspinal drive of specific spinal locomotor circuitries because c-Fos expression in neurons normally increases when after the control of spinal locomotor circuitries’ circuitry control is lost [41,42]. Consistent with previous studies, c-Fos expression in nuclei (c-Fos + nuclei) was observed in all rats in levels L1-L5 (Figure 5A-C) [43]. In addition, the number of c-Fos + nuclei tended to be lower at the L1-L5 levels in SM-345431-treated SCI rats than in control SCI rats, but this difference did not reach statistical significance (Figure 5B). Interestingly, the combined treatment group showed a statistically significant decrease in the number of c-Fos + nuclei compared to control SCT rats at the L4 and L5 levels (Figure 5B, P < 0.01). We also compared the rostral (L1 + L2) and caudal (L4 + L5) segments of the lumbar enlargement to examine the overall effects of combinatorial treatment (Figure 5C). Compared to the intact group (normal rats), control SCT rats showed a significant increase in c-Fos + nuclei counts in the caudal segments of the lumbar enlargement (L4-5), which is consistent with previous reports (Figure 5C, P < 0.01) [24]. On the other hand, after combined treatment, the caudal segments of the lumbar enlargement showed significantly decreased c-Fos + nuclei counts (Figure 5C, P < 0.01) as compared to control SCT rats. The number of c-Fos + nuclei also tended to be decreased after SM-345431 treatment alone, although this difference was not statistically significant (Figure 5C). Thus, specific spinal locomotor circuitries, especially in the caudal sections of the lumbar enlargement, were considerably reinforced by combined treatment and, to a lesser extent, by SM-345431 treatment alone.

Synapsin-1, a widely used presynaptic marker, has been used to examine activity-dependent synaptic plasticity and synaptic function [39,40]. In comparison to the control and SM-345431 groups, a statistically significant increase in synapsin-1 expression was observed at the lumbar enlargement level (Figure 5E-N) (P < 0.01 compared to the control SCT group; P < 0.05 compared to the SM-345431 group) in the combined treatment group. No statistically significant difference was observed between the SM-345431 group and the control SCT group (P > 0.05). These results indicated that, while SM-345431 treatment alone had a limited effect, SM-345431 treatment combined with treadmill training significantly reinforced synaptic plasticity and function at the lumbar enlargement level.

Taken together, these data suggest that reinforcement of specific spinal locomotor circuitries and motor learning occurred in the lumbosacral circuits of adult rats after combined treatment. These effects were also observed, to a lesser extent, after SM-345431 treatment alone.

### SM-345431 promoted motor functional recovery and combined treatment enhanced this recovery

**Figure 6:**
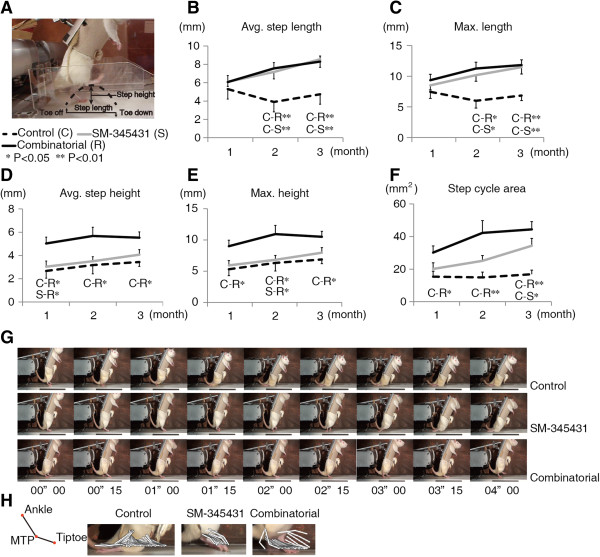
**Detailed kinematic analysis of hindlimb motor performance on the treadmill. (A)** Representative image of the analysis and a schematic diagram of the step parameters. Collected kinematic data from each group showing the average step lengths **(B)**, maximum step lengths **(C)**, average step heights **(D)**, maximum step heights **(E)** and step cycle areas **(F)**. All of these data were recorded and analyzed using a robotic device (Rodent Robot 3000; Robomedica Inc.). Each kinematic analysis was performed at 70% BWS and a speed of 1 cm/s over 1 min. C represents the control group, S represents the SM-345431 group, and R represents the combined treatment group. *P < 0.05, **P < 0.01. C-S* indicates that a statistically significant difference was observed between the control and SM-345431 groups at P < 0.05. Statistical analyses were performed using one-way ANOVA with Bonferroni *post hoc* analyses. Data are presented as the means ± S.E.M. **(G)** Representative sequential pictures of steps taken during the first 4 s of the analysis (BWS: 70%, speed: 3 cm/s). **(H)** Kinematic characteristics of the first step taken in each group. SM-345431 treatment enhanced motor function recovery on the treadmill, especially in terms of the step length parameters. Combined treatment further enhanced the motor function recovery, especially in terms of the step height and step cycle area parameters.

Next, we investigated the hindlimb motor functions of rats while they walked on a treadmill (induced by a robotic device) using kinematic analysis. In the control SCT rats, hindlimb motor functions showed almost no recovery throughout the period of our experiments (3 months). The SCT rats were unable to take any steps on a treadmill even 3 months post-injury, whereas the treated animals showed some degree of motor function recovery. We performed Bonferroni *post hoc* analyses to examine the differences in step length and step height between the groups (Figure 6A) and found that motor function was significantly better in SM-345431 rats than in control SCT rats (Figure 6B-C). Furthermore, SM-345431 treatment with treadmill training further improved motor performance as compared to no treatment (Figure 6B,C,D,E) or SM-345431 treatment alone (Figure 6D,E). To evaluate motor functions in greater detail, we examined the “step cycle area”, which was calculated by multiplying the average step length and average step height as previously described [44] (Figure 6F). This parameter includes elements of both the horizontal and vertical step planes and can thus be regarded as a 2-dimensional evaluation of each step taken. We again performed Bonferroni *post hoc* analyses to statistically compare the differences between the groups. In the SM-345431-treated rats, a statistically significant enhancement of the step cycle area was observed in comparison to the control SCT rats (P < 0.05 at 3 months). Furthermore, the combined treatment resulted in an even greater improvement in step cycle area when compared to control SCT rats (P < 0.05 at the first month, P < 0.01 at the second month, P < 0.01 at the third month). As previously described [20], the control SCT rats could not take any steps on the treadmill even at 3 months post-injury (Figure 6G,H and Additional file 1). Without extensive treadmill training, the effects of SM-345431 treatment were moderate and did not result in plantar step walking, even with the BSS (Figure 6G,H and Additional file 1). However, when combined with extensive treadmill training, SM-345431 treatment led to considerably enhanced motor function recovery; all rats exposed to the combined treatment achieved continuous plantar step walking on a treadmill with the BSS for at least 30 min (Figure 6G,H and Additional file 1).

Specifically, SM-345431 treatment improved locomotor function on a treadmill after SCT particularly in terms of the step length parameters (Figure 6B,C: average and maximum step lengths). Regarding the step height parameter, the effect of SM-345431 treatment was moderate and statistically insignificant (Figure 6D,E). However, when SM-345431 treatment was combined with extensive treadmill training, greater enhancement was observed, and this enhancement extended to the step height parameter (Figure 6D,E; average and maximum step heights). Furthermore, the incremental effects of the treatments over time on motor function performance, specifically on step height and step cycle area, did not reach a plateau by the end of the experimental period (3 months post-injury). At this time, the incremental effects tended to be more robust in the SM-345431 treatment group than in the combined treatment group (Figure 6F: step cycle area). Interestingly, we also discovered a statistically significant correlation between the number of c-Fos + nuclei and motor function (step cycle area) within each group; the c-Fos + nuclei counts were inversely correlated with the extent of functional motor recovery (Figure 5D; r = -0.501, P < 0.05).

**Figure 7:**
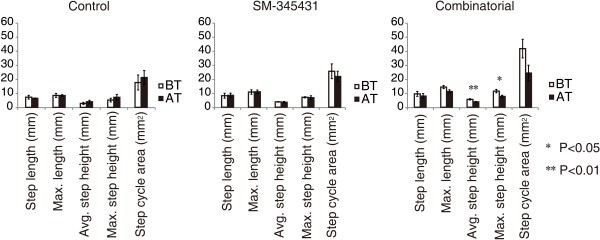
**Detailed kinematic analyses of hindlimb motor performance on a treadmill before and after re-transection.** Detailed kinematic analyses of hindlimb motor performance on a treadmill before and after re-transection were performed and analyzed using a robotic device (Rodent Robot 3000; Robomedica Inc.). BT represents before re-transection; AT represents after re-transection. Although none of the step parameters in the control group showed significant changes, the step cycle area parameters were attenuated in the SM-345431 group after re-transection. Further attenuation of the step ability parameters was observed in the combined treatment group after re-transection. *P < 0.05 **P < 0.01. Statistical analyses were performed using *t*-test analyses. Data are presented as the mean ± S.E.M.

We next examined the extent to which the regenerated axons at the lesion sites contributed to motor function recovery in each group. We performed re-transection of the initial lesion site 12 weeks after the initial injury, and we also performed the kinematic analysis before and after the re-transectioning procedure. None of the step parameters changed significantly in the control group, whereas the step cycle area tended to be attenuated in the SM-345431 group after re-transection (Figure 7). Interestingly, greater attenuations of step ability were observed in all analyzed parameters in the combined treatment group after re-transection, and some of these parameters (step height) were statistically significant (Figure 7, average step height: P < 0.01; maximum step height: P < 0.05). Thus, the regenerated axons at the lesion site contributed, at least partially, to the enhancement of motor function recovery.

## Discussion

With clinical applications in mind, we first sought to find an appropriate and efficient way to deliver our semaphorin3A inhibitor to transected spinal cords to reestablish useful motor function after SCI. For this purpose, we developed a novel DDS and tested its therapeutic potential *in vivo*. Furthermore, we tested whether specific rehabilitation had the capability to reinforce motor function in the SCT model after treatment with the semaphorin3A inhibitor. Our results can be briefly summarized as follows. First, our newly developed DDS utilizing silicone sheets provided continuous and stable drug delivery and therefore demonstrated potential clinical application. Second, motor function recovery was considerably enhanced by combining SM-345431, the novel semaphorin3A inhibitor, with treadmill training, and this combined treatment enabled paralyzed rats to perform continuous plantar step walking on a treadmill with a BSS.

Osmotic mini pumps have been widely used for drug delivery in animal models of SCI [20,45]. While these pumps are excellent for controlling the drug release dosage and the accurate positioning of drug delivery, this method is too invasive for use in most patients. Therefore, we developed a new silicone matrix preparation as a novel DDS and showed that this novel DDS provided stable and continuous release of SM-345431 throughout the experimental period. In addition, because SM-345431 shows a better stability in a silicone sheet than SM-216289 and approximately the same semaphorin3A inhibitory activity as SM-216289, we used SM-345431 instead of SM-216289 in the present study. As a result, significantly enhanced axonal regeneration and motor function recovery were observed after SM-345431 treatment, which is consistent with our previous study using SM-216289 and osmotic mini pumps [20]. Therefore, this novel DDS demonstrated strong potential for future clinical use.

In this study, we examined the effects of a new semaphorin3A inhibitor, SM-345431, on axonal regeneration and motor function recovery after SCT. Our data indicated that SM-345431 administration using the novel DDS was effective in promoting axonal regeneration and motor function recovery *in vivo*. However, consistent with the results of our previous study using SM-216289, these effects were moderate [20]. More importantly, we found that motor function recovery was significantly enhanced by combining SM-345431 with extensive treadmill training (Figure 6). This combined strategy enabled paralyzed rats to perform continuous plantar step walking on a treadmill with a BSS. Previous work has also demonstrated that intact or injured axons in descending tracts and propriospinal circuits can undergo spontaneous anatomical and physiological remodeling after SCI [46,47] to allow the relay of information through endogenous spinal circuits. Subsequently, the novel propriospinal relay connections bypass the injury epicenter to induce supraspinal control and some degree of motor function recovery. This phenomenon has also been observed following the irreversible interruption of long descending tracts in mice [48]. Because SM-345431 treatment without extensive treadmill training had limited effects on anatomical reconstruction in the lumbar enlargement (Figure 5), we speculate that the enhanced motor function recovery observed after SM-345431 treatment resulted mainly from axon regeneration and limited anatomical reconstruction. Enhanced axonal regeneration possibly led to anatomical and physiological remodeling at the lesion site and improved motor function recovery, which is consistent with our previous findings using SM-216289 administered via osmotic mini pumps [20].

Interestingly, while axonal regeneration was not further enhanced by combining SM-345431 treatment with extensive treadmill training (Figure 2), motor function recovery was significantly enhanced (Figure 6). Previous work demonstrated that limited spontaneous axonal regeneration occurs after SCI and that only some of the new axons are useful for motor function recovery [49]. Thus, axonal regeneration alone may not result in sufficient motor function recovery unless these regenerated axons are appropriately connected. We therefore hypothesized that the regenerated axons induced by semaphorin3A inhibitor treatment may exhibit substantially improved rewiring, in terms of the formation of appropriate connections, following extensive treadmill training. Indeed, motor function recovery was enhanced significantly by combined treatment, as paralyzed rats that received combined treatment were able to perform continuous plantar step walking on a treadmill with a BSS (Figure 6). Re-transectioning experiments further suggested that the regenerated axons at the lesion site and their rewiring contributed to the enhancement in motor function recovery observed in the treatment groups (Figure 7). We also observed that some regenerated axons, such as 5-HT-positive raphe-spinal tract axons, penetrated into the epicenter of the lesion (Figure 2), and these regenerated axons may have contributed to the reorganization of spinal circuitry at the lesion site and enhanced motor function recovery in the treatment groups [20]. Interestingly, we observed greater attenuation of motor function after re-transection in the combined treatment group (Figure 7). Taken together, these data indicate that the regenerated axons achieved substantial rewiring to the appropriate targets at the lesion site, which was associated with the rehabilitation performed in the combined treatment group [21].

Moreover, the enhanced motor function recovery was only partially attenuated after re-transection in the treatment groups. The results of these re-transection experiments in the treatment groups, especially in the combined treatment group, suggested that other factors, such as reorganization of the lumbar spinal circuitry, also contributed to the enhancement of motor function recovery. We also performed re-transection experiments in animals that only received extensive treadmill training (without SM-345431 treatment), and in these animals, only minimum attenuation of motor performance after re-transection was observed (data not shown). This finding further supports the mechanism proposed above. Taken together, it is possible that specific rehabilitation enhances not only the rewiring of the regenerated axons but also the reorganization of the lumbar spinal circuitry [43]. Furthermore, in the combined treatment group, it is possible that the effect of specific rehabilitation on the reorganization of the lumbar spinal circuitry was the main contributor to the enhancement of motor function recovery in the early stage, while the effect on rewiring of the regenerated axons was the main contributor to the enhancement of motor function recovery during the later stage.

The results of c-Fos immunohistochemistry (Figure 5) showed that extensive treadmill training induced plastic changes at a level caudal to the injured site. Interestingly, step ability on the treadmill was significantly correlated with decreases in the numbers of c-Fos-positive nuclei at a level caudal to the lumbar enlargement (Figure 5D). Therefore, enhanced motor function recovery could be partially the result of anatomical reconstruction of the lumbar spinal cord, which is thought to be the location of CPGs [50]. It is now widely accepted that supraspinal drive of CPGs is required for locomotor function [51] and that the intrinsic plasticity of CPGs allows some spontaneous motor function recovery even in the absence of significant axonal regeneration [24,52]. Therefore, it is possible that axon regeneration moderately enhanced motor function recovery in the SM-345431 treatment group, while rewiring of the regenerated axons and CPG activation was achieved by combining the SM-345431 treatment with treadmill training.

Which segment of the lumbar enlargement is most important for improved walking ability on a treadmill? Reportedly, rhythmic bursts are related to flexor activity at the L2 level and to extensor activity at the L5 level [50]. Treadmill training induces plastic changes in the transmission of group I pathways to extensors that consequently support the recovery of weight-supported motion while standing [53]. The present study showed that combined treatment significantly altered c-Fos expression at the L4 and L5 levels (Figure 5B-C) and improved step height (Figure 6D-E) and continuous plantar step walking on the treadmill with a BSS (Figure 6G and Additional file 1). However, while SM-345431 alone improved step ability, this improvement did not lead to continuous plantar step walking (Figure 6). Therefore, axon regeneration alone might have only limited effects on the spinal cord extensor pool, which could explain the limited motor function recovery of SCT rats after SM-345431 treatment alone. However, the combined treatment may have reestablished the function of the extensor pool by rewiring the regenerated axons and remodeling spinal circuits. As a result, important motor functions, such as continuous plantar step walking (on a treadmill with a BSS), may have been reestablished in SCT adult rats in the combined treatment group. Thus, combining SM-345431 treatment with specific rehabilitation is a reasonable and promising approach to the treatment of SCI.

Other possible mechanisms underlying motor function recovery could include remyelination and angiogenesis. We noticed that, with increases in afferent sensory input, the step lengths of the SM-345431 groups while walking on the treadmill exhibited a linear and gradual improvement throughout the experimental period. The animals treated with SM-345431 showed enhanced remyelination at the lesion site (Figure 4E-J), which could be relevant to a recent study reporting the effects of semaphorin3A on myelination [36]. In general, myelination significantly increases conduction velocity (sometimes up to 100-fold [54]), which results in increased motor function. Thus, remyelination after SM-345431 treatment may have also partially contributed to the enhancement of motor function recovery on the treadmill. Angiogenesis also plays an important role in reducing secondary damage and enhancing tissue repair after SCI, and the extent of angiogenesis correlates with the extent of axon regeneration after SCI [33]. Angiogenesis was significantly enhanced after SM-345431 treatment alone, although combined treatment did not further enhanced this effect statistically (Figure 4A-D). Therefore, angiogenesis may also have contributed to motor function recovery. Interestingly, at the end of the experimental period (3 months post-injury), the incremental effects of the treatment on motor performance, specifically in terms of step height and step cycle area, tended to be more robust in the SM-345431 treatment group than in the combined treatment group (Figure 6D-F). These data indicate that combined treatment may also have expedited motor function recovery and decreased the overall time needed for recovery.

## Conclusions

Collectively, our data demonstrate that the administration of SM-345431 via a novel DDS utilizing silicone sheets significantly enhanced axonal regeneration, remyelination and angiogenesis, thereby promoting motor function recovery after SCT in adult rats. Additionally, combining SM-345431 with extensive treadmill training resulted in improved motor function recovery that included continuous plantar step walking on a treadmill with a BSS. This comprehensive effect of combined treatment presumably resulted from the reinforcement of spinal networks in the caudal spinal stump and the rewiring/refinement of regenerated axons. Thus, combining semaphorin3A inhibitor treatment with extensive treadmill training has great potential as a new treatment for SCI. In addition, this study highlights the importance of combining treatments that promote axon regeneration with specific and appropriate rehabilitations that promote rewiring for the effective treatment of SCI.

## Methods

### Overall experimental outline

Rats were randomly divided into the following three experimental groups: 1) untrained + placebo, 2) untrained + SM-345431 and 3) trained + SM-345431. SCT was performed, and SM-345431 or placebo was administered at the lesion site via the newly developed DDS, which is described in detail below. Starting 1 week post-injury, treadmill training commenced with a BSS. Kinematic tests were performed monthly for 3 months after SCT using a rodent robotic device (Rodent robot 3000, Robomedica Inc.) that primarily assessed the performance of plantar stepping on a treadmill. Treadmill training was continued throughout the experimental period.

### Animals and surgical procedures

A total of 53 adult female Sprague-Dawley rats (200-250 g, 10-12 weeks old) were used in this study (3 rats died during the experimental period and were excluded from the statistical analysis). All procedures were approved by the experimental animal care committee of Keio University, School of Medicine and Murayama Medical Center (approval #12-8). All rats were anesthetized with an intraperitoneal injection of ketamine (100 mg/kg)/xylazine (10 mg/kg). The spinal cord at the level of the T10 lamina was exposed by T10 laminectomy, and the dorsal dura mater was opened. The exposed spinal cord was cut along the inner edge of the vertebra with a sharp micro-scissor. Two more cuts were made at the gap of the transected spinal cord by a scalpel to ensure total transection. SM-345431 or placebo was administered to the transected site via the newly developed DDS as described in detail below (Figure 1G-I). After these procedures, the back muscles and skin were closed. Rats were kept warm in an incubator (37°C) after surgery. To prevent dehydration in the rats, 10 ml of saline was subcutaneously injected daily until day 7. Ampicillin (0.4 g/kg) was also injected intramuscularly daily to prevent infection until day 7. The bladder was evacuated manually until autonomous emptying of the bladder was achieved. The re-transection procedure was performed at the same level as the primary SCT (15 rats total; 5 rats from each group). Kinematic data were recorded using similar procedures prior to re-transection surgery (on the same day) and on the day following re-transection surgery. For CST tracing, 10% BDA was injected as follows. Nine weeks after SM-345431 or placebo administration, BDA (10000 MW, Molecular Probes) was injected into six different sites of the sensorimotor cortices of the rats under general anesthesia (site 1: 2.0 mm lateral, 0 mm to bregma; site 2: 2.0 mm lateral, 2 mm posterior to bregma; site 3: 2.0 mm lateral, 4 mm posterior to bregma; site 4: 4 mm lateral, 0 mm to bregma; site 5: 4 mm lateral, 2 mm posterior to bregma and site 6: 4 mm lateral, 4 mm posterior to bregma). For each site, injections were performed at two different depths (1.2 mm and 1.6 mm), and 3 μl of 10% BDA was injected at a rate of 0.15 μl/min using a micro-injector. Three weeks after the BDA injection, rats were sacrificed and used for immunohistochemistry.

### Growth cone collapse assay and collagen co-culture assay

The growth cone collapse assay and collagen co-culture experiments were performed as previously described [20]. To examine the effects of SM-345431-silicone, small pieces (2 × 1 × 0.3 mm; approximately 1 mg containing 1 μg of SM-345431) of the SM-345431-silicone or control-silicone were placed in a collagen gel adjacent to E8 chick DRGs and COS7 cell aggregates, as shown in Figure 1C.

### Drug delivery system

A novel matrix silicone preparation was developed to allow continuous drug delivery at the site of injury. The amount of drug released from this matrix silicone preparation *in vitro* was measured as described in Figure 1. Matrix silicone sheets (0.3 mm thick) containing SM-345431 were trimmed into 3-mm-square pieces to fit into the opened dura. After SCT, one piece of silicone sheet was placed on the transected spinal cord gap so that it could act on the spinal cord directly. Silicone sheets of the same size that did not contain SM-345431were used for the control group.

### Training protocol

A robotic device (rodent robot 3000, Robomedica Inc.) [55] was used to train the SCT rats. Briefly, the device consisted of a computer-controlled BSS, two lightweight robotic arms and a treadmill with variable motorized speeds. The ankles of the hindlimbs of rats were held with a pair of releasable rope cuffs, which were then secured to robotic arms to track ankle trajectory in the horizontal and vertical directions. A computer-controlled body support arm was used to control the load that was applied to the hindlimbs and to maintain body equilibrium. Rats were secured in a cloth vest and attached to the body support arm with a hook-and-loop fabric. Hard rope cuffs were attached to the hindlimbs of rats with the robotic arm during training.

In our pilot study, we found that it was possible to train spinal cord-transected rats soon after SCT via voluntary walking evoked by sensory input. Additionally, improvements in motor performance were more obvious when the treadmill training was initiated at earlier time points after SCT. Therefore, training was initiated as early as 1 week after SCT. The fixed parameters were set at 50% body weight support (BWS), 20 min/day and 5 days/week. The animals were adapted to the training via increasing velocity; a velocity of 1 cm/s was used in the first week, and then the velocity was increased by 2 cm/s every 2 weeks for the first 2 months after injury (i.e., 1 cm/s to 3 cm/s to 5 cm/s). In the first week of training, rats frequently did not adapt to the acceleration of the treadmill, and this resulted in dragging of the hindlimbs. Once the rats dragged their hindlimbs and stopped walking on the treadmill, a trainer brought their bodies back to the original walking position. The step ability of SCT rats on the treadmill improved gradually over the course of the first 2 months following injury. However, this improvement was attenuated at time points later than 2 months after injury. Hence, at the time points later than 2 months after injury, the velocities of the treadmill were adjusted to 5 to 9 cm/s according to the improvement observed in the hindlimb motion of the rats.

### Detailed motor function analysis using kinematics

To evaluate the locomotor capability of SCT rats in detail, the aforementioned robotic device was employed. Each robotic arm tracked the two-dimensional movement of the ankle, and the trajectory of the ankle movement was then recorded on a computer for kinematic analyses. Not all the rats were able to walk by themselves on the treadmill by the last time point of the experiment. Therefore, when performing the tests, the degree of BWS and treadmill speed were titrated to obtain the maximum walking performance on the treadmill. As a result of this titration, the behavioral tests were performed at 70% BWS and a treadmill velocity of 1 cm/s each month after SCT. The duration of testing was 1 min per rat to minimize training effects during testing. The methods of previous reports [44,56] were followed with slight adaptations. Briefly, the ankle trajectory in each plane was recorded by the robotic arm and a computer. Then, the toe off (TO) and paw contact (PC) events in each step cycle were identified using Rodent Robot 3000 software. All kinematic characteristics were obtained when TO and PC were identified; as a result, parameters such as the duration phase, the swing phase of the step cycle and the length and height of the step were calculated. The number of animals used in these behavioral tests was 32 (control: n = 9, SM-345431: n = 12, combined: n = 11).

### Immunohistochemistry

Twelve weeks after SCT, rats were deeply anaesthetized by an intraperitoneal injection of 14% chloral hydrate and then perfused intracardially with 4% paraformaldehyde (PFA) in 0.1 M phosphate-buffered saline (PBS). The spinal cord tissues were dissected and post-fixed in 4% PFA (24 h) and placed in 10% sucrose in 0.1 M PBS (24 h) followed by 30% sucrose in 0.1 M PBS (24 h). All the rats other than 3 rats died during the experimental period were used for the histological analysis. Segments of spinal cords were embedded in Optimal Cutting Temperature compound (Tissue Tek) and stored at -80°C. Frozen spinal cord tissues were cut with a cryostat into 20-μm-thick sections. For diaminobenzidine (DAB) staining, sections were washed with 0.1 M PBS and then presoaked for 30 min in 0.03% H_2_O_2_ with methanol. After an additional presoak in TNB (0.10 M Tris-HCl, 0.15 M NaCl, 0.5% BMP) for 60 min, sections were incubated at 4°C with rabbit anti-GAP43 (1:300; Millipore), mouse anti-rat RECA-1 (1:500; Serotec) or rabbit polyclonal anti-synapsin-1 (1:300; Chemicon) for 24 h. Subsequently, the sections were washed in 0.1 M PBS and incubated with biotinylated secondary antibodies (1:1,000; Jackson Immunoresearch) for 1 h. Next, the sections were washed and then incubated with an avidin-biotin complex (ABC) (Vectastain Elite ABC Kit, Vector Laboratories) in TNB (1:100) and visualized using DAB (Sigma). Sections were rinsed in PBS, dehydrated using ethanol and xylene and cover-slipped with permount. To identify 5-HT-positive axons that penetrated into the scar tissue area after the treatment, we used a previously described double-staining method [28]. 5-HT was visualized using goat anti-serotonin (5-HT) antibody (1:500; ImmunoStar) and DAB with nickel-glucose oxidase, which produced a black stain. Sections were washed and then incubated with rabbit monoclonal anti-glial fibrillary acidic protein (GFAP; 1:1,000; BD Bioscience Pharmingen) and visualized with DAB, which produced a brown stain. Following these procedures, we identified the range of the scars and quantified the number of 5-HT-positive axons that penetrated into the scar tissue area. To evaluate the status of axonal myelination, immunofluorescent double staining was performed using rabbit anti-GAP43 (1:1,000; Millipore) and rat monoclonal anti-MBP (1:50; Abcam) antibodies. Immunohistochemical analysis for c-Fos in spinal neurons was performed using procedures similar to those previously described [24,41,43]. Briefly, rats were trained using the aforementioned training method of continuous hindlimb bipedal stepping. After 45 min of continuous hindlimb bipedal stepping at 3 cm/s with 50% BWS with a hard nylon rope attachment, rats were allowed a 60-min rest. Subsequently, the rats were anesthetized and perfused intracardially with 4% PFA in PBS. After perfusion, the spinal cords were dissected, post-fixed for 24 h at 4°C and cryoprotected in 30% sucrose in PBS for 3 days. The L1-L5 segments were mounted and frozen, and 20-μm-thick axial sections were cut using a cryostat. All sections were pretreated with 0.03% H_2_O_2_ and methanol for 30 min and then incubated with rabbit polyclonal anti-c-Fos antibody (1:200; Santa Cruz Biotechnology) for 24 h (at 4°C). Subsequently, the sections were washed in 0.1 M PBS and incubated in biotinylated secondary antibody (1:1,000; goat antibody against rabbit; Jackson ImmunoResearch) for 1 h. The remaining procedures were identical to those performed for DAB staining, as described above. All images were obtained using either an Axioskop 2 Plus microscope (Zeiss) for DAB staining or a LSM510 confocal microscope (Zeiss) for immunofluorescent staining.

### Electron microscopic analysis

For electron microscopic analysis, rats from the 3 groups were sampled 72 days after injury. Rats were perfused with 4% PFA in PBS, and the spinal cords were dissected and post-fixed with 2.5% glutaraldehyde overnight at 4°C. After 90 min of fixation with 0.5% osmium tetroxide, the spinal cords were dehydrated with ethanol, acetone and QY1 and then embedded. Ultrathin sections at the epicenter of the lesion sites were prepared at a thickness of 80 nm and stained with uranyl acetate and lead citrate for 15 and 12 min, respectively. The sections were observed with a transmission electron microscope (JEOL model 1230), and images were acquired using Digital Micrograph 3.3 (Gatan Inc.).

### Quantitative immunohistochemistry analyses

Immunohistochemical image analyses were performed for all sections of each animal using microscopy, and quantitative analyses were performed by an examiner who was blind to the identities of the animals. Each value is presented as the average value per section (unless otherwise indicated). The number of animals used for quantitative analysis of each staining set ranged from 15 to 21 (5 to 7 animals per group). To quantify the area of GAP-43-positive axons, 5-HT-positive axons and RECA-1-positive vessels, sagittal sections of the spinal cord at the injury site (approximately 1.2 cm in length) were scanned with a CCD camera (DXC-390; Sony). Pictures of the sagittal sections at 1 mm to 3 mm rostral and 1 mm to 3 mm caudal from the injury epicenter were captured for quantitative analyses. The images were analyzed with a Micro Computer Imaging Device (MCID; Imaging Research Inc.). Threshold values were maintained at constant levels for all analyses. 5-HT axons that penetrated into the scar tissue were counted manually. For image analysis, c-Fos-positive (c-Fos+) nuclei from all sections was superimposed onto Molander’s cytoarchitectonic maps of the rat thoracic and lumbosacral cord [57]. The expression of synapsin-1 was examined within lamina IX of the L1-L5 segments of the spinal cord using transverse sections and DAB staining. For the quantification of BDA tracing, we followed the methods reported previously [58,59]. The number of CST-positive axons at each distance from the lesion was divided by the number of CST-positive axons at the level of C1 for standardization.

### Statistical analyses

For statistical analyses, one-way analyses of variance (one-way ANOVA) and Bonferroni *post hoc* tests were primarily employed to determine significance. Significance was determined using P-values, and the data are presented as the means ± S.E.M. For the analysis of 5-HT immunostaining, data were analyzed with the Kruskal-Wallis H test. Behavioral data after re-transection were analyzed with t-tests.

## Competing interests

H. Okano is a scientific consultant of San Bio, Inc; Eisai Co Ltd; and Daiichi Sankyo Co Ltd.

K. Kikuchi, A. Sano, M. Maeda, A. Kishino and T. Kimura are employed by Dainippon Sumitomo Pharma Co Ltd.

## Authors’ contributions

LZ and SK performed the experiments and wrote the manuscript. KK, AS, MM, SS, and MM performed the experiments. SK, AK, YT, ML, TK, HO and MM designed the study. All authors read and approved the final manuscript.

## Supplementary Material

Additional file 1 **Video Representative movies of the *detailed *kinematic *analysis of hindlimb motor performance *on a treadmill at the end of the experimental period.** Plantar step walking with a BSS was not observed in control group animals (A). Limited plantar step walking with a BSS was observed in SM-345431 treatment group animals (B). Significantly enhanced plantar step walking with a BSS was observed in the combined treatment group animals. All animals in the combined treatment group continued plantar step walking with a BSS for at least 30 min (C). Click here for file
